# Editorial

**Published:** 1869-10

**Authors:** 


### Editorial.

Female Students and Physicians.—A recent letter from a correspondent in Europe closes with the following report from the *Gazette de Hospiteau:*—"Decidedly, the medical profession in France can no longer be considered the exclusive property of the male sex. Among the students who have passed the most brilliantly their examinations to the Faculty of Medicine are included three ladies—one French, one Russian, and one American. The latter has proved herself to possess a solid knowledge of anatomy, and dissections, pathology, and operative surgery."

It thus appears, that female students have not only gained admittance to the best hospitals and medical schools of Europe, for instruction, but they have finally been admitted to regular examinations and graduation, by the highest medical tribunal in France.

Is it not time that the position of female medical students should be more definitely settled in this country? At present, they are admitted freely to clinical instruction in the hospitals of most of our large cities, but are denied admission into the colleges, or to become regular candidates for graduation. If, in despite of the latter obstacle, one of them persists, until she finally gets a degree, from some school in this country or Europe, she is freely recognized in practice, and consulted with by members of the profession of the highest standing; and if she can get a hospital or infirmary for the treatment of women and children started, any number of respectable physicians are ready to lend their names to make up the usual list of *consulting* physicians, surgeons, obstetricians, etc. Now, if it is right thus to admit them to clinical instruction, in hospitals, consult with them in private practice, and associate our names with theirs, as attendants on public institutions, where is the consistency or propriety of shutting them out of the colleges? Certainly, there is no more indelicacy in mingling the sexes in the lecture room of a college than around the operating table of a public hospital. We are not disposed to encourage women to study medicine, simply because its practice is not a business suited to her nature, or which she can follow successfully, without ignoring some of the most important objects of her own creation. To do the work of a general practitioner, she must be ready in the darkness of night, as well as at noonday; in storm as well as sunshine—at all hours, to ride over the country, or traverse the streets or alleys of cities, all of which she cannot do without stultifying her own social instincts, and avoiding the performing of some of the highest duties required of her by her Creator. And, yet, it is doubtless true, that there is now, and always will be, a very few females who are so constituted, mentally and physically, that they will exhibit a persistent disposition to study and practise medicine. These, if well educated, could be made very useful members of the profession, and find honorable and remunerative employment in the practise of special departments of the profession, in our more populous cities. Hence, we think, it would be much better, if the medical colleges should open their doors to them, and give them the same opportunities, and hold them to the same full requirements as the other sex.

---

Chicago Medical College—Medical Department of the North-Western University.—The next annual course of instruction in this institution commences on Monday, the 4th day of October. The general introductory lecture will be given in the College Hall, on Monday evening, by H. W. Boyd, M.D., Professor of Descriptive Anatomy.

We feel no hesitation in saying, that the student of medicine, who is earnestly desirous of thoroughly educating himself in all the departments of medical science and practise, can find no college in this country more complete in its arrangements, more comprehensive in its curriculum, or more reliable in the faithful execution of what it promises, than this. There is already a good class attending the clinical instruction in Mercy Hospital, the new building for which is rapidly approaching completion.

---

Archives de Physiologie Normale et Pathologique. Publiees par MM. Brown-Sequard, Charcot, Vulpian.—The number of this very valuable French journal for September and October is promptly on table. Its contents are varied and highly interesting.

---

Pharmacist.—We have received the September number of the *Pharmacist and Chemical Record* of this city, containing the proceedings of the recent National Pharmaceutical Convention, but too late to enable us to notice its contents in this number. We were absent from the city during the sessions of the Convention, or we should have been happy to have personally witnessed its proceedings.

---

Canadian Medical Association.—The second annual session of this Association was recently held in Toronto. We enjoyed the pleasure of attending its sessions, and forming the personal acquaintance of many of its members. We had prepared an abstract of such part of its proceedings as would interest our readers, but want of space will compel us to postpone its insertion until our next number.

### Mortality for the Month of August, 1869:—

| | |
|---|---|
| Accident, drowned | 14 |
| " burns, kerosene | 4 |
| " by fall | 4 |
| " railroad | 3 |
| " run over by wagon | 2 |
| Anasarca | 1 |
| " and fever scarlet | 1 |
| Apoplexy | 4 |
| Ascitis and enlargement of liver | 1 |
| Asthma | 2 |
| " and consumption | 1 |
| Births, premature | 26 |
| " still | 47 |
| " tedious | 2 |
| Bowels, inflammation, | 4 |
| Brain, congestion of | 8 |
| " compression of, | 1 |
| " disease of | 3 |
| " inflammation | 6 |
| " malformation | 1 |
| " softening of | 1 |
| Cachexia, malarial | 1 |
| Cancer of superior maxillary bone | 1 |
| Childbirth | 1 |
| Cholera infantum | 328 |
| Cholera morbus | 7 |
| Convulsions | 86 |
| Consumption | 40 |
| Convulsions, puerperal | 5 |
| Croup | 8 |
| " diphtheretic | 1 |
| " membranous | 2 |
| Cyanosis | 1 |
| Debility | 3 |
| " general | 1 |
| Delirium tremens | 1 |
| Diarrhœa | 81 |
| " chronic | 4 |
| " whooping-cough | 2 |
| Diphtheria | 12 |
| Dropsy | 3 |
| Dysentery | 38 |
| " chronic | 1 |
| " typhoid | 2 |
| Encephalitis | 5 |
| Endo-carditis | 1 |
| Entero-colitis | 5 |
| Enteritis | 12 |
| Epilepsy | 1 |
| Exhaustion | 1 |
| Erysipelas | 1 |
| Fever, congestive | 1 |
| " puerperal | 7 |
| " scarlet | 53 |
| " " malignant, | 3 |
| " typhoid | 19 |
| " typhus | 2 |
| Gastritis | 3 |
| Gastro-enteritis | 4 |
| Hemorrhage, internal, | 1 |
| Heart, disease of | 3 |
| " organic disease of | 1 |
| " valvular " | 2 |
| " fatty degeneration of | 1 |
| Hemipligia | 2 |
| Hepatitis, acute | 2 |
| Hernia, umbilical | 1 |
| Hydrocephalus | 17 |
| " acute | 7 |
| Inanition | 10 |
| " and diarrhœa | 1 |
| Intestinal obstructions | 1 |
| Jaundice | 1 |
| Kidneys, Bright's dis. | 1 |
| Liver, atrophy of | 1 |
| " disease of | 1 |
| " abscess of | 1 |
| " inflammation of, | 1 |
| Lungs, con estion of | 2 |
| Mouth, canker sore | 1 |
| Measles | 15 |
| " and diarrhœa | 1 |
| " and teething | 2 |
| Meningitis | 12 |
| " cerebro-spinal, | 7 |
| Meningitis, tubercular, | 2 |
| Manslaughter | 3 |
| Neuralgia | 1 |
| Old age | 6 |
| Otitis | 1 |
| Paralysis | 4 |
| Peritonitis | 3 |
| " puerperal | 3 |
| Phrenitis | 1 |
| Pleurisy | 3 |
| Pneumonia | 9 |
| " typhoid | 1 |
| Poisoned, unknown | 1 |
| Pyæmia | 1 |
| " from complicat'd fracture of leg | 1 |
| Rheumatism, cardiac | 1 |
| Scrofula | 3 |
| " of hip joint and gastritis | 1 |
| " of intestines | 1 |
| Small-pox | 1 |
| Septæmia | 1 |
| Spine, fracture of | 1 |
| Syphilis | 1 |
| Stomach, cancer of | 2 |
| " hemorrhage of | 1 |
| " scirrhosis of | 1 |
| Sun-stroke | 3 |
| Suicide | 7 |
| Tabes mesenterica, | 39 |
| Teething | 26 |
| " and convulsions, | 6 |
| Throat, ulcerated sore, | 1 |
| " putrid sore | 1 |
| Uremia | 1 |
| Vitality, deficient | 1 |
| Whooping-cough | 24 |
| " and teething | 1 |
| Womb, contraction of, | 1 |
| " inflammation of, | 1 |
| Wound to foot from fall | 1 |
| Total | 1070 |

### COMPARISON.

Deaths in Aug., 1869,—1070 | Deaths in Aug., 1868,— 945 | Increase,—125

Deaths in July, 1869,— 815 | Increase,—255

### AGES.

| | |
|---|---|
| Under 1 | 488 |
| 1 to 3 | 313 |
| 3 to 5 | 44 |
| 5 to 10 | 25 |
| 10 to 20 | 24 |
| 20 to 30 | 53 |
| 30 to 40 | 45 |
| 40 to 50 | 31 |
| 50 to 50 | 21 |
| 60 to 70 | 14 |
| 70 to 80 | 8 |
| 80 to 90 | 2 |
| 90 to 100 | 1 |
| Unknown | 1 |
| Total, | 1070 |

| | | |
|---|---|---|
| Males, 548 | Females, 522 | Total, 1070 |
| Single, 950 | Married 120 | Total, 1070 |
| White, 1062 | Colored, 8 | Total, 1070 |

NATIVITY.

| | | | | | |
|---|---|---|---|---|---|
| Austria | 1 | Denmark, | 2 | Poland | 1 |
| Atlantic Ocean | 3 | England, | 8 | Scotland, | 5 |
| Bohemia, | 7 | France, | 1 | Sweden, | 29 |
| Bavaria | 1 | Germany, | 75 | Wales | 3 |
| Canada, | 10 | Holland | 5 | Unknown, | 4 |
| Chicago, Native, | 197 | Ireland, | 41 | | |
| Chicago, Foreign, | 549 | Italy | 1 | Total, | 1070 |
| U. S., other parts, | 109 | Norway, | 18 | | |

MORTALITY BY WARDS FOR THE MONTH.

| Wards. | Mortality. | Pop. in 1868. | One death in | Mortality. | |
|---|---|---|---|---|---|
| 1 | 16 | 9,094 | 568⅓ | Accidents, | 27 |
| 2 | 18 | 13,074 | 726⅓ | Bridewell | 1 |
| 3 | 39 | 15,076 | 384 | County Hospital, | 21 |
| 4 | 62 | 17,796 | 287 | Home for Friendless, | 6 |
| 5 | 95 | 16,033 | 168 7-9 | Immigrants, | 32 |
| 6 | 64 | 13,083 | 204½ | Jewish Hospital, | 1 |
| 7 | 104 | 25,492 | 245 1-9 | Mercy Hospital, | 1 |
| 8 | 103 | 15,813 | 153½ | Marine Hospital, | 2 |
| 9 | 53 | 19,297 | 364 1-10 | Manslaughter, | 3 |
| 10 | 24 | 12,925 | 538½ | Protestant Orphan Asylum, | 1 |
| 11 | 29 | 14,340 | 494½ | St. Joseph Orphan Asylum, | 7 |
| 12 | 135 | 17,485 | 129½ | Poisoned, unknown | 1 |
| 13 | 55 | 11,164 | 203 | Convent of Good Shepards | 1 |
| 14 | 62 | 14,839 | 239⅓ | Suicide, | 7 |
| 15 | 58 | 21,078 | 363 3-7 | | |
| 16 | 42 | 15,465 | 368 1-5 | Total, | 1070 |

---

Resolutions of Respect upon the Death of Dr. F. O. Earle.— *Whereas*, the Chicago Medical Society has been deprived of one of its members, by the death of Dr. F. O. Earle.

*Resolved*, that we lament his loss. Although he had been with us but a brief time, we had learned to respect him for his gentlemanly deportment and professional ability. Although quite young in the profession, he had given promise of more than ordinary ability, and could look forward to a noble and useful future in the profession of his choice. We cannot but feel that the close application and devotion to the labor of the profession, during his stay among us, had much to do in determining his early death, which seemed too soon. The profession has lost an earnest worker. To his family and relatives we may be permitted to express our sincere sympathy.

*Resolved*, that a copy of these be sent to the family, and that they be published in the medical journals of this city, and in the *Medical Record* of New York.

R. G. Bogue, M.D.
T. D. Fitch, M.D.
A. H. Foster, M.D.

